# The Surgical Treatment Principles of Atlantoaxial Instability Focusing on Rheumatoid Arthritis

**DOI:** 10.1155/2015/518164

**Published:** 2015-07-26

**Authors:** Yu-Tung Shih, Ting-Hsien Kao, Hung-Chuan Pan, Hsien-Te Chen, Hsi-Kai Tsou

**Affiliations:** ^1^Department of Neurosurgery, Jen-Ai Hospital, No. 483 Dong Rong Road, Dali, Taichung 41265, Taiwan; ^2^Functional Neurosurgery Division, Neurological Institute, Taichung Veterans General Hospital, 1650 Taiwan Boulevard, Section 4, Taichung 40705, Taiwan; ^3^Graduate Institute of Medical Science, National Defense Medical Center, No. 161, Section 6, Minquan E. Road, Neihu District, Taipei 11490, Taiwan; ^4^Department of Acupressure Technology, Jen-Teh Junior College of Medicine, Nursing and Management, No. 79-9, Sha-Luen-Hu, Xi Zhou Li, Hou-Loung Town, Miaoli County 35664, Taiwan; ^5^Faculty of Medicine, School of Medicine, National Yang-Ming University, No. 155, Section 2, Linong Street, Taipei 11266, Taiwan; ^6^School of Chinese Medicine, College of Chinese Medicine, China Medical University, No. 91, Hsueh-Shih Road, Taichung 40402, Taiwan; ^7^Department of Orthopaedic Surgery, China Medical University Hospital, No. 91, Hsueh-Shih Road, Taichung 40402, Taiwan; ^8^Department of Rehabilitation, Jen-Teh Junior College of Medicine, Nursing and Management, No. 79-9, Sha-Luen-Hu, Xi Zhou Li, Hou-Loung Town, Miaoli County 35664, Taiwan

## Abstract

*Object.* This retrospective review was conducted to determine the surgical treatment principle for rheumatoid arthritis (RA) patients with atlantoaxial instability (AAI). *Methods.* Thirteen patients with AAI, including 5 RA patients, received preoperative computed tomography- (CT-) based image-guided navigation system (IGS) in C1 lateral mass-C2 pedicle screw-rod system fixation (LC1-PC2 fixation). These 13 patients were analyzed for 52 screws inserted into C1 and C2. We defined these patients as non-RA group (8 patients, 32 screws) and RA group (5 patients, 20 screws). The neurological status for RA group was evaluated using the Ranawat classification. The causes of AAI, surgical indications, complications, surgical method revolution, and CT-based navigation application are discussed. *Results.* None of the 13 patients expressed neurological function deterioration. The non-RA group screw accuracy was 100%. In the RA group, 1 RA patient developed left C2 screw loosening at 1^+^ months after operation due to screw malposition. The screw accuracy for this group was 95%. *Conclusions.* Higher intraoperative surgical complication rate was described in RA patients. Preoperative CT-based IGS in LC1-PC2 fixation can provide good neurological function and screw accuracy results. However, for higher screw accuracy in RA patients, intraoperative CT-based IGS application may be considered.

## 1. Background and Introduction

AAI is characterized by excessive movement between the atlas and axis. It is notorious for nuchal pain and neural compression. Early recognition of the progressive neurological symptoms for early surgical intervention is an important predictor for good recovery [1, 2]. Various surgical methods were applied in AAI.

RA patients may have bone erosion and osteoporosis due to rheumatoid synovitis and medication. Therefore, besides trauma, infection, congenital disease, and postirradiation status, RA is another important risk factor for AAI [3]. In addition, patients with comorbid rheumatoid and spine pathology have been shown to have higher wound and implant-related complications [4]. LC1-PC2 system was used in RA patients for higher fusion rate. In addition, more preop evaluation, surgical planning, and postop care should be considered in RA patients.

## 2. Methods

### 2.1. Patient Classification

Thirty-five patients with AAI were treated between April 2004 and September 2014 by one surgeon at one institute. Eighteen patients (51.4%) were female and 17 (48.6%) were male (mean age, 55.3 years, range 21–77 years). All patients were divided into trauma group (19 patients; 54.2%), RA group (6 patients; 17.1%), degenerative osteoarthritis (4 patients; 11.4%), movement disorder group (1 patient, 2.8%), symptomatic Os odontoideum (1 patient, 2.8%), osteomyelitis (1 patient, 2.8%), previous implant failure group (1 patient with previous titanium cable wire fixation and autogenous iliac bone fusion; 2.8%), and patients with unknown cause (2 patients, 5.7%) (Table 1).

### 2.2. Preop Survey

**Figure 1:**
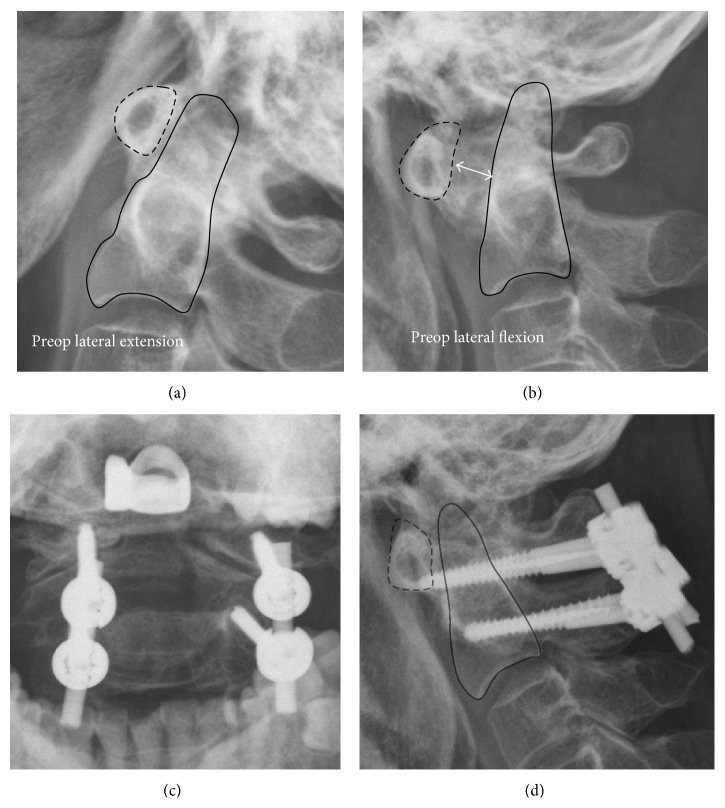
Preop dynamic lateral radiographs revealed AAI ((a) and (b)). The atlantodental interval 6 mm in flexion position (b) is measured (double-headed arrow). Postop radiographs revealed atlantoaxial fusion with LC1-PC2 system ((c) and (d)).

**Figure 2:**
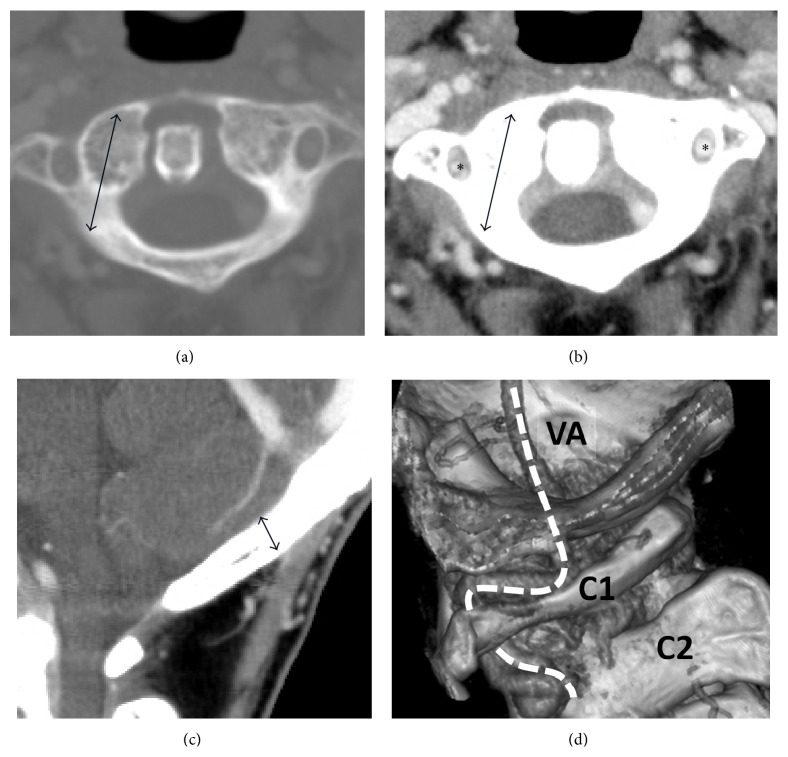
Preop reconstructive computed tomography for choosing pedicle screw length and diameter (a), knowing the screw and vertebral artery relationship (*asterisk*) (b), choosing occiput screw length (c), and the vertebral artery (*dashed line*) direction (d).

Radiographs in AP view, lateral flexion-extension view, and open-month view were checked for bone structure and stability (Figures 1(a) and 1(b)). Before surgery, 128-slice spiral CT scanning (Philips, iCT 256) was carried out. Patients were placed supine for spinal CT axial scanning, and the data were recorded in DICOM format in the computer. The scanning conditions were 140 kV voltage and 171 mA electric current. The scanning parameters included image matrix 512 × 512, slice thickness 0.9 mm, pitch 0.49, and reconstruction slice thickness of 1 mm. Careful preoperative study of this CT scan with 3D reconstruction including the occiput was acquired to check occipital bone thickness, vertebral artery, and diameter and length estimation of the lateral mass or transpedicle screw before operation (Figure 2). In the cases with CT-based IGS, the images were then transferred into the navigation system (BrainLAB Vector Vision Navigation System).

### 2.3. Operation Methods

One patient received transoral partial odontoidectomy and decompression prior to posterior approach with LC1-PC2 fixation for chronic C1-2 subluxation with pseudotumor and spinal cord compression. One patient underwent transoral biopsy prior to LC1-PC2 fixation owing to difficult osteomyelitis or tumor differential diagnosis by neuroradiologist. One patient received C1-2 Halifax interlaminar clamp with autogenous iliac bone fusion. Six patients received anterior odontoid screw fixation. Five patients received occipitocervical fusion with screw-rod system (4 patients O-C2-C3, 1 patient O-3-4-5; one of these 4 patients received revision surgery as replacement for loosening occiput Y-plate with screw-rod system).

**Figure 3:**
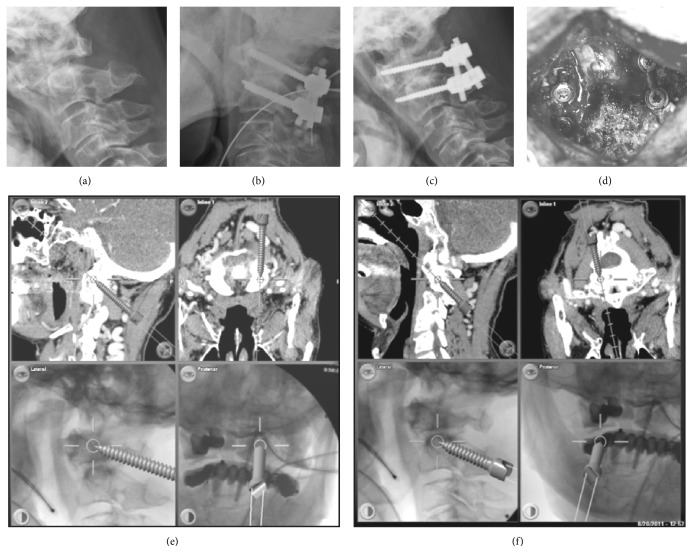
Preop lateral radiograph revealed AAI (a). Postop radiographs revealed fixation after operation ((b) and (c)). The operative photo showed LC1-PC2 system (d). The intraoperative CT navigation guided technique for placement screw for C1 (e) and C2 (f).

Twenty-three patients received LC1-PC2 fixation (Figures 1(c) and 1(d)) (1 of the 23 patients was in post-Gallie wiring and grafting techniques with cable wire breakage). Three-dimensional (3D) assessment with a preoperative CT-based IGS was applied in 13 of these 23 patients since February 2012 (Figures 3(e) and 3(f)). There were 6 RA patients enrolled in this study. The one who presented cranial settling and received occipitocervical junction fusion with a screw-rod system was excluded from the screw accuracy analysis. These 13 patients, including 5 RA patients, were analyzed for 52 screws inserted into C1 and C2. We defined these patients as non-RA group (8 patients, 32 screws) and RA group (5 patients, 20 screws).

## 3. Results

None of the 13 patients who received preoperative CT-based IGS in LC1-PC2 fixation expressed neurological function deterioration. Five patients had a history of RA. Their neurological status was evaluated using the Ranawat classification (Table 2). Two of these 5 patients were Ranawat class IIIA, another patient was Ranawat class IIIB, and the remaining two patients were classified as Ranawat classes I and II.

**Figure 4:**
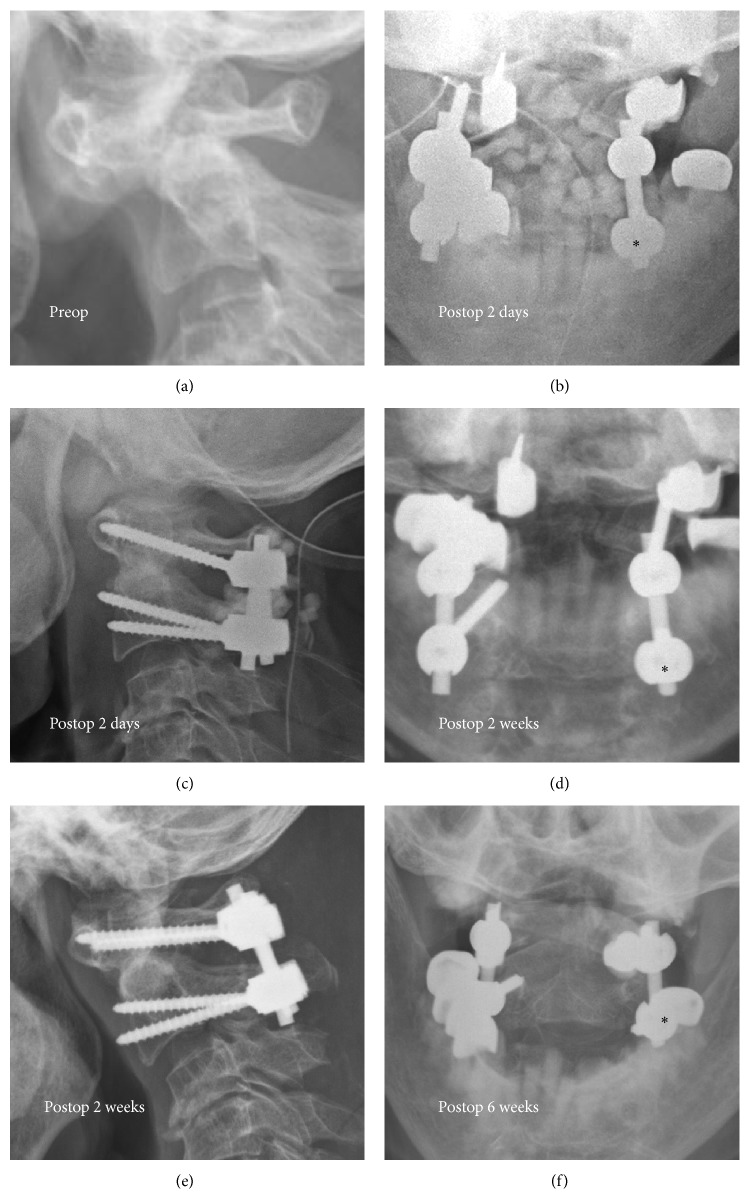
Preop lateral radiograph revealed AAI (a). Second day postop radiographs showed malposition of left C2 screw (*asterisk*) ((b) and (c)). Two weeks postop radiographs for follow-up of the fusion condition ((d) and (e)). Six weeks postop open mouth radiograph revealed loosening of left C2 screw (f).

Satisfactory C1-2 screw placement and atlantoaxial reduction were achieved in all patients except one RA patient with left C2 screw malposition. This patient developed left C2 screw loosening at 1^+^ months after operation due to screw malposition during surgery (Figure 4). The patient hesitated at reopening surgery owing to uneventful outcome from the screw loosening.

These 13 patients (52 screws for C1 and C2) received preoperative CT-based IGS for LC1-PC2 fixation. Of the 32 screws inserted in the non-RA group (8 patients), 32 screws were in the correct position. Of the 20 screws inserted in the RA group (5 patients), 19 screws were in the correct position. The non-RA group screw accuracy was 100%. The C1 and C2 screw accuracy in the RA group was 95%.

Two of these 23 patients who received LC1-PC2 fixation (including “virtual fluoroscopy” and navigation system) suffered from occipital neuralgia. There were no vertebral artery (VA) injuries during the operations and no neurological deterioration after surgery related to the procedure.

One patient received O-C2-C3 Y plate and pedicle screw-rod fixation system. Three years later occiput Y-shaped plate screw dislodgement was found in radiograph. A revision operation was performed.

One major complication occurred in one quadriparesis patient in trauma group due to chirotherapy who received an atlantoaxial fixation using a LC1-PC2 system. The pain was relieved and muscle power much improved in all four limbs after the operation. We weaned the patient from the ventilator 1 day after operation. However, suffocation and cardiac arrest occurred on the 6th day after operation. With emergency cardiopulmonary resuscitation the patient's vital signs recovered. However, dull consciousness with ventilator support persisted. Three years after operation she died due to cardiopulmonary failure.

## 4. Discussion

AAI is characterized by disproportionate movement between the atlas and axis due to either bony or ligamentous abnormality. AAI may occur after trauma, upper respiratory infection or infection following head and neck surgery, inflammatory disease as rheumatoid arthritis, or congenital disease. The most common cause for AAI is trauma. Tiu KL reported that irradiation-related delayed healing, higher infection risk, and osteonecrosis may result in atlantoaxial instability [5].

Cervical spine involvement occurs in over half of patients with RA. The atlantoaxial joint is often affected in patients with RA [6]. Atlantoaxial subluxation causes the odontoid process or the posterior arch of the atlas to impinge on the spinal cord. Spinal cord and C2 root compression can result in such symptoms as myelopathy, occipital pain, and nuchal pain in RA patients [7]. Medication, rehabilitation, and surgical intervention have their roles in different conditions. However, when it comes to intractable pain, progressive neurological deficits, or progressive instability, surgical intervention is indicated [8].

Rheumatoid cervical disease usually develops within 2–10 years of RA [9]. A cohort study with 161 patients described the natural course of cervical lesions in RA. Ninety-two patients (57%) had upper cervical involvement, which progressed into anterior atlantoaxial subluxation, vertical subluxation, and both. Neural involvement occurred in 10 patients. In 7 of these 10 patients vertical subluxation of the atlas was responsible for the neural deficit [10]. In a cohort of 55 rheumatoid cervical patients who received surgery after myelopathy deteriorated to Ranawat class IIIB patients. The early postoperative mortality rate was high (12.7%). Only 14 patients (25.5%) were judged to have had a favorable outcome as determined by an improvement to Ranawat class I or II [11]. Mikulowski et al. reported postmortem findings in 104 rheumatoid patients and found that 11 deaths were associated with cervicomedullary compression from atlantoaxial dislocation [12]. The cervicomedullary compression may cause serious sequelae, including paresis, hypertonia, delayed motor milestones, and respiratory compromise. Because of the poor natural history, it is believed that earlier surgical intervention, before the development of vertical translocation, permanent neurological damage, and spinal cord atrophy, is necessary in RA patients.

More than 80% of RA could be detected with cervical spine involvement by radiology modalities [13]. In basilar invagination cases with RA, alar ligament and tectorial membrane relaxation or disruption developed. The odontoid process then migrates upward. It is radiologically defined by the amount of protrusion of the tip of the odontoid process by more than 5 mm beyond McGregor's line.

AAI is defined if atlantodental interval is greater than 3 mm in adults and greater than 5 mm in children. The atlantodental interval is the distance between the posterior aspect of the anterior atlas ring and the anterior aspect of the odontoid process (Figures 1(a) and 1(b)). Other papers pointed that the posterior atlantodental interval has been found to be a more sensitive tool for evaluating neurologic injury because it reflects the potential spinal canal. The decrease in potential space for the spinal cord increases the risk for spinal cord compression. The posterior atlantodental interval, also known as space available for spinal cord (SAC), critical lower limit is 13 mm, which has a 97% sensitivity to predict paralysis [14, 15].

Furthermore, image studies for the preoperative survey include CT angiography and MRI of the cervical spine. Both of these studies are helpful for planning the diameter, length, and trajectory of screws to avoid vertebral arteries and neural structures injuries during screw insertion (Figure 2).

The cessation of various rheumatoid medications before the operation is another issue for reducing surgical complications. For example, nonsteroidal anti-inflammatory drugs should be discontinued 3 to 5 half-lives before surgery. Perioperative corticosteroids stress doses should be given. Methotrexate should be discontinued for 6 to 8 weeks because it will increase the infection rate and affect bone healing. Biological agents (tumor necrosis factor-*α* and interleukin-1 antagonists) should be stopped preoperatively and held until 14 days after surgery to avoid the risk of opportunistic infections [16].

Operating on the atlantoaxial complex has always posed a challenge to the surgeon because of the complex anatomy and biomechanics of this spine region. Historically, Gallie wiring and grafting techniques were used for AAI [17], which were further modified by Brooks and Jenkins [18]. Three variants of lateral mass screws have been described by An, Magerl, and Roy-Camille. Goel and Laheri described plate and screw fixation for atlantoaxial subluxation which was further modified by Harms and Melcher to posterior C1 lateral mass and C2 pedicle or pars screws [19, 20]. In addition, in case of AAI with basilar invagination, anterior decompression with occipitocervical fusion and decompressive laminectomy was suggested [21]. Due to the high biomechanical strength, posterior atlantoaxial fixation using lateral mass screws at C1 in combination with pedicle screws at C2 has become popular [19]. In the C2 pedicle screw insertion procedure, complications from screw penetration into spinal canal and VA injury can occur. If one VA is hypoplastic, it presents lethal complications when the dominant VA is ruptured. It is more risky to insert a pedicle screw in patients with narrow C2 pedicle. Therefore, even though the pedicle screws have been shown to have the highest pullout strength, some authors did not recommend routine use due to a higher risk for vertebral artery injury [22]. In addition, occipital neuralgia was a frequent complication related to posterior atlantoaxial fixation. It is due to surgical manipulation during preparation of the C1 screw entry point and impingement of C2 root by C1 screw. Gautschi et al. reported that among the clinically relevant complications related to posterior atlantoaxial fixation, postoperative C2 neuralgia is the most frequent problem (9.8%) [23]. To avoid occipital neuralgia complications, C2 root sacrifice is performed by some surgeons. The C2 root resection is also believed to achieve safe and wide exposure in performing C1-2 instrumented fixation. However, numbness occurs in approximately 12% of patients who received C2 root resection; a result that may be intolerable to certain patient populations [24, 25].

The poor bone quality of RA patients makes both intra- and postoperative periods more complex. Gallie or Brook wiring and bone grafting methods were used to fuse the atlantoaxial joint. This method achieved a lower fusion rate than other fusion methods in the general population. Case reports on wire-graft fusion complications due to C1 posterior arch fracture were described in RA patients [26]. The Magerl technique, known as C1-C2 transarticular screw fixation with posterior wiring, developed for higher fusion rate. Kuroki and colleagues reported that LC1-PC2 fixation is more stable than transarticular screw fixation [27]. Therefore, we propose that LC1-PC2 fixation provides more stability and less late complication rate in RA patients. However, when performing the C2 pedicle screw fixation, the VA may be injured. This is a disastrous complication. Few studies have compared the risk for VA injury in patients with and without RA. Masahiko demonstrated that RA is a significant risk factor for a narrow C2 pedicle, and narrowing of the C2 pedicle is elevating the risk for VA injury in RA patients. Therefore, in patients with RA, thorough preoperative evaluation of the bone architecture is very important for avoiding inadvertent injury to the VA [28]. Another paper reported that the cervical pedicle screw perforation rate was higher in spine tumors (16.7%), RA (37.5%), and destructive spondyloarthropathy patients [29]. Therefore, closer attention should be paid to the atlantoaxial complex in patients with RA.

Preoperative cervical CT is useful for bone architecture and surgical planning. Preoperative screw trajectory could be evaluated for avoiding inadvertent VA injury. Using 3D assessment with a CT-based IGS, the axial cut planning for the instrumented levels presents extreme benefit in determining the proper screw trajectory for the safety of adjacent neural and vascular structures during the operation. A systematic review including 18 cohort studies and 2 randomized controlled trials revealed that there is a significantly lower risk of pedicle perforation for navigated screw insertion compared with nonnavigated insertion for all spinal regions [30]. Linhardt et al. demonstrated increased pullout strength in pedicle screws placed with computer-assisted techniques compared with screws placed with conventional techniques [31]. Nottmeier et al. believed that using the 3D image guidance system planning function allows larger diameter screws to be placed, resulting in screws being placed in a more medial trajectory than standard techniques [32]. It is believed that 3D assessment with a CT-based IGS can avoid vascular and neural injury and also can enhance the screw-rod system stability. However, severe cervical pedicle screw malposition can occur even with 3D navigation. Ishikawa et al. recorded that, even with 3D navigation, prevalence of cervical pedicle screw perforations was 18.7% in their study [33]. In our practical experience, the possibility of screw malposition by preoperative CT-based IGS may be related to a change in body posture from the supine position during preoperative CT scanning to the prone position during the operation. We adjusted the patient's neck posture using the Mayfield pin-fixation device for better atlantoaxial reduction in the operation room prior to the posterior approach. These are the reasons why inaccurate screw placement occurred in preoperative CT-based IGS, particularly in RA patients with severe AAI. A large retrospective comparative outcome analysis [34] revealed excellent accuracy in preoperative and intraoperative CT-based IGS; there was a statistically significant advantage for intraoperative group. Intraoperative CT-based IGS application may provide higher screw accuracy in this patient group.

## 5. Conclusion

Higher intraoperative surgical complication rate was described in RA patients. Preoperative CT-based IGS in LC1-PC2 fixation can provide good neurological function and screw accuracy results. However, for higher screw accuracy in RA patients, intraoperative CT-based IGS application may be considered. Although the CT-based IGS surgical technique was used to decrease the complication rate and improve instrument biomechanical stability, advanced techniques, surgical experience, and anatomy knowledge are required to decrease the screw malposition rate.

## Figures and Tables

**Table 1:** Indications for atlantoaxial instability surgery.

Indications	Patients (*n* = 35)
Fracture	19
Rheumatoid arthritis	6
Degenerative osteoarthritis	4
Movement disorder	1
Symptomatic Os odontoideum	1
Osteomyelitis	1
Previous implant failure	1
Unknown	2

**Table 2:** Ranawat classification of neurological deficit.

Ranawat classification	

Class I	No neural deficit

Class II	Subjective weakness, dysesthesias, and hyperreflexia

Class IIIA	Objective weakness and long-tract signs; patient remains ambulatory

Class IIIB	Objective weakness and long-tract signs; patient no longer ambulatory
